# Synergistic Effects of Amitraz and Dinotefuran on Honey Bee Health: Impacts on Survival, Gene Expression, and Hypopharyngeal Gland Morphology

**DOI:** 10.3390/ijms26146850

**Published:** 2025-07-17

**Authors:** Mojtaba Esmaeily, Tekalign Begna, Hyeonjeong Jang, Sunho Kwon, Chuleui Jung

**Affiliations:** 1 Department of Plant Medicals, Gyeongkuk National University, Andong 36729, Republic of Korea; mesmaeily18@gmail.com (M.E.); tekalign12@gmail.com (T.B.); jhj971008@naver.com (H.J.); andong171@naver.com (S.K.); 2 Agricultural Research Institute, GyeongKuk National University, Andong 36729, Republic of Korea

**Keywords:** *Apis mellifera*, neonicotinoids, anti-*Varroa* acaricides, side effects, synergistic effects

## Abstract

Honey bees (*Apis mellifera*) are major pollinators, playing a critical role in global food production, biodiversity, and ecosystem stability. However, their populations are increasingly threatened by multiple interacting stressors, including pesticide exposure. Among these, agricultural insecticides and anti-*Varroa* acaricides such as dinotefuran and amitraz can persist in hive matrices, resulting in chronic and combined exposure. This study investigates the low lethal (LC_10_ and LC_30_) effects of these compounds, individually and in combination, on honey bee survival, immune function, oxidative stress responses, detoxification pathways, and hypopharyngeal gland morphology. Both pesticides negatively affected honey bee health at low lethal concentrations, with dinotefuran showing higher toxicity. Exposure led to the reduced survival, suppression of vitellogenin expression, and dysregulation of genes related to antioxidant defense, immunity, and detoxification. Additionally, high concentrations of dinotefuran and its combination with amitraz impaired hypopharyngeal gland morphology. Notably, co-exposure resulted in synergistic toxic effects, exacerbating physiological damage beyond individual treatments. These findings emphasize the potential risks of combined exposure to agricultural and beekeeping pesticides. A more comprehensive risk assessment and stricter regulations are urgently needed.

## 1. Introduction

Honey bees (*Apis mellifera*) are indispensable pollinators, playing a pivotal role in global food production and ecosystem sustainability [1]. As eusocial insects, they contribute not only through pollination but also by providing essential hive products such as honey, beeswax, and royal jelly. Honey bees, along with other non-*Apis* pollinators, help pollinate nearly 80% of the world’s flowering plants [2]. Beyond their agricultural value, honey bees play a crucial ecological role in maintaining biodiversity and supporting natural ecosystems [1,3]. Despite their significance, honey bee populations are experiencing alarming declines due to a combination of biotic and abiotic stressors, including parasites, pathogens, habitat loss, pesticide exposure, and climate change [4,5,6,7]. These stressors often interact synergistically, exacerbating colony losses and making their decline a complex, multifactorial issue [8,9,10,11]. Addressing these challenges is essential for ensuring the long-term stability of both agricultural productivity and natural ecosystems.

Honey bees are frequently exposed to pesticides, particularly those used for *Varroa destructor* control within colonies [12]. To combat this parasitic mite, beekeepers commonly apply synthetic acaricides such as amitraz (formamidine), coumaphos (organophosphate), and fluvalinate (pyrethroid) [12,13]. While these chemicals are approved for use in honey bee colonies and, when applied correctly, do not cause immediate harm, even low concentrations can have low lethal effects on bee physiology, neurology, metabolism, and behavior [14,15]. Additionally, residues of these acaricides can accumulate in hive components such as honey, wax, and pollen, resulting in chronic exposure for both adult and immature bees [11,16,17].

Beyond *Varroa* control, agricultural pesticides, particularly neonicotinoids, pose a significant threat to honey bee populations. These chemicals have been closely linked to behavioral disruptions, weakened antioxidant defenses, and suppressed immune function, making bees more susceptible to pathogens and further exacerbating colony losses [7,18,19,20,21,22]. Neonicotinoids have been widely used since the 1990s for controlling piercing–sucking pests such as aphids and leafhoppers, largely due to their systemic activity and high efficacy [23]. However, their extensive and prolonged application has raised concerns about unintended effects on non-target organisms, particularly honey bees. Growing evidence suggests that even a low lethal exposure to neonicotinoids can significantly disrupt key aspects of honey bee biology, including reproduction, longevity, navigation, foraging efficiency, and overall colony development [24,25,26].

As previously mentioned, honey bees are exposed to various pesticides both within the hive and in agricultural landscapes. Among these, amitraz, an acaricide widely used for *Varroa destructor* control, and dinotefuran, a neonicotinoid insecticide, are frequently detected in hive matrices due to their extensive application in beekeeping and crop protection [27,28,29,30]. While the individual effects of amitraz and dinotefuran on honey bees have been widely studied [27,28,29,30], and numerous studies have examined synergistic interactions between pesticides with different modes of action [31,32,33,34], their combined impact remains largely unexplored. This is particularly important given that amitraz and dinotefuran are commonly used for distinct purposes in both beekeeping and agriculture. Studies specifically investigating their co-exposure effects on honey bee survival, immune function, oxidative stress responses, and detoxification pathways are scarce. Given the widespread use of these chemicals, the likelihood of honey bees encountering both pesticides simultaneously is high, underscoring the need for further research into their potential synergistic toxicity.

To address this gap, we investigated the low lethal (LC_10_ and LC_30_) impact of amitraz and dinotefuran, both individually and in combination, on honey bee survival, immune function, oxidative stress responses, and hypopharyngeal gland morphology. We analyzed key molecular markers, including antimicrobial peptides (as indicators of immune function), antioxidant enzymes (catalase and superoxide dismutase, which counteract oxidative stress), and detoxification enzymes (cytochrome P450s and glutathione S-transferases, which facilitate pesticide metabolism). Additionally, we examined the expression of vitellogenin, a well-known marker involved in oxidative stress regulation and longevity, and assessed structural alterations in the hypopharyngeal gland morphology, which are essential for brood care. By integrating survival analysis, gene expression profiling, and morphological assessments, this study provides a comprehensive evaluation of the risks associated with pesticide co-exposure in honey bees.

## 2. Results

### 2.1. Toxicity Assessment of Amitraz and Dinotefuran in Honey Bees

The results indicate that amitraz has a significantly higher LC_50_ (500.027 ppm) than dinotefuran (3.413 ppm), indicating that dinotefuran is considerably more toxic to honey bees at lower concentrations. This trend is also evident in the LC_10_ and LC_30_ values, confirming that dinotefuran exhibits higher toxicity to honey bees compared to amitraz under the conditions tested (Table 1). In this study, LC_10_ and LC_30_ (lethal concentrations causing 10% and 30% mortality, respectively) were used to represent low lethal exposure levels. These concentrations were subsequently used to designate treatment groups, where A_10_ and A_30_ refer to amitraz at LC_10_ and LC_30_, and D_10_ and D_30_ refer to dinotefuran at LC_10_ and LC_30_. Combinations such as A_10_D_30_ or A_30_D_30_ denote co-exposure to both pesticides at the respective concentrations.

### 2.2. Effect of Amitraz, Dinotefuran, and Their Mixture on Honey Bee Survival

**Figure 1:**
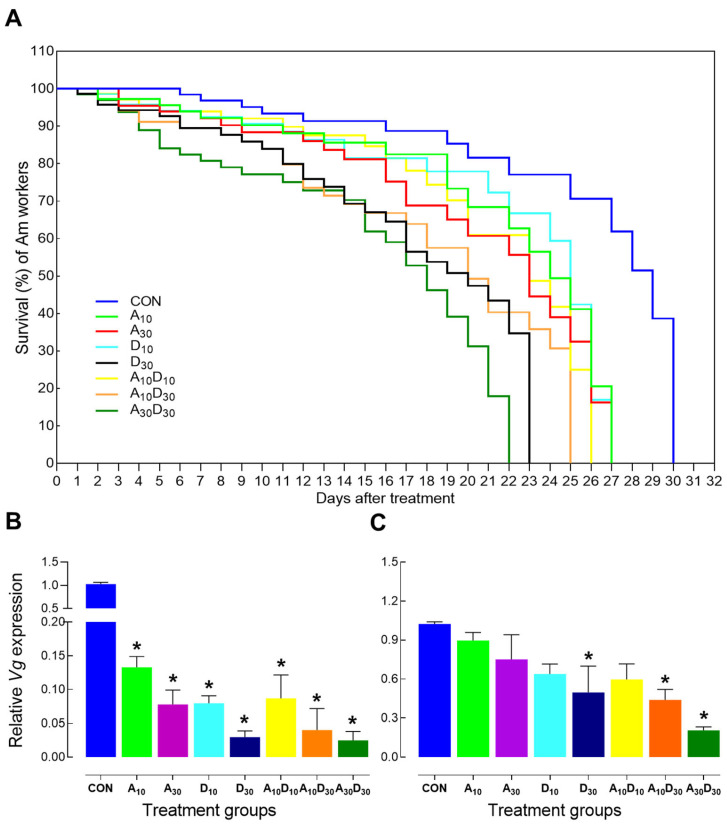
Survival analysis and vitellogenin (Am-Vg) expression in *A. mellifera* workers exposed to insecticides. (**A**) Kaplan–Meier survival curves showing the effects of LC_10_ and LC_30_ concentrations of amitraz (A_10_, A_30_), dinotefuran (D_10_, D_30_), and their mixtures (A_10_D_10_, A_10_D_30_, A_30_D_30_) on worker bee survival. Each treatment was replicated 10 times with 10 bees per replicate, and mortality was recorded until all bees per group had died. (**B**,**C**) Relative expression of Am-Vg in *A. mellifera* workers at 24 h (**B**) and 7 days (**C**) post-treatment. Expression analysis was conducted using three biological replicates per treatment. Error bars represent standard deviation of the mean (SD). Asterisks indicate significant differences compared to the control group (* *p* < 0.05, Tukey’s HSD test).

The survival analysis of *A. mellifera* workers revealed significant differences among treatments (Figure 1). The control group maintained the highest survival throughout the experiment, confirming that mortality was treatment-induced. Exposure to amitraz (A_10_, A_30_) or dinotefuran (D_10_, D_30_) resulted in a dose-dependent decrease in survival, with higher concentrations (A_30_ and D_30_) leading to more rapid mortality. While acute toxicity was evident at higher concentrations, the long-term effects were also pronounced, as surviving bees exhibited progressively declining survival rates over time, suggesting that a low lethal exposure may lead to physiological impairments or delayed toxicity, ultimately reducing lifespan. The combination treatments (A_10_D_10_, A_10_D_30_, A_30_D_30_) led to an accelerated decline in survival compared to individual insecticides, indicating a potential synergistic effect. Notably, A_30_D_30_ exhibited the steepest decline, suggesting that co-exposure to high concentrations of amitraz and dinotefuran enhances toxicity beyond their individual effects. Additionally, vitellogenin (*Am-Vg*) expression was significantly affected by pesticide exposure at both 24 h and seven days’ post-treatment (Figure 1B,C). A single exposure to amitraz or dinotefuran led to a significant reduction in *Am-Vg* expression after 24 h compared to the control (*F* = 569.66, *p* < 0.0001), with higher concentrations (A_30_ and D_30_) showing a more pronounced decline (Figure 1B). This indicates that even short-term exposure to these insecticides can disrupt physiological functions related to longevity and stress resistance in honey bees. Moreover, after seven days, the suppression of *Am-Vg* expression was even more evident, particularly in the treatments with dinotefuran LC_30_ (D_30_, A_10_D_30_, A_30_D_30_), which exhibited the most significant reductions (*F* = 50.2, *p* < 0.0036) (Figure 1C). The A_30_D_30_ group displayed the lowest expression levels, suggesting a potential synergistic effect of amitraz and dinotefuran in impairing vitellogenin synthesis. Since vitellogenin plays a crucial role in immunity, oxidative stress regulation, and colony maintenance, this sustained decline highlights the long-term physiological impact of pesticide exposure, even after a single treatment.

### 2.3. Effects of Amitraz, Dinotefuran, and Their Mixture on Stress-Related Gene Expression

**Figure 2:**
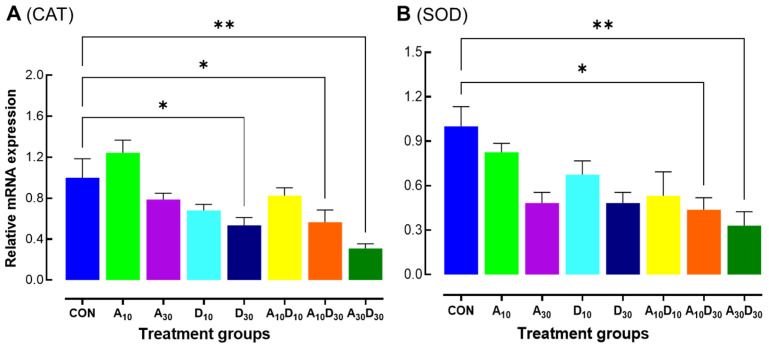
Low lethal effects of amitraz, dinotefuran, and their mixture on oxidative stress-related gene expression in newly emerged *A. mellifera* workers. Relative mRNA expression levels catalase (Am-CAT) (**A**) and superoxide dismutase (Am-SOD) (**B**) were analyzed in honey bees 24 h after exposure to LC_10_ and LC_30_ concentrations of amitraz (A_10_, A_30_), dinotefuran (D_10_, D_30_), and their mixtures (A_10_D_10_, A_10_D_30_, A_30_D_30_). Three independent biological replicates were used per condition. Error bars represent standard deviation of the mean (SD). Asterisks indicate significant differences compared to the control group (* *p* < 0.05, ** *p* < 0.01, Tukey’s HSD test).

The relative mRNA expression levels of catalase (CAT) and superoxide dismutase (SOD) were analyzed to evaluate oxidative stress responses following exposure to amitraz and dinotefuran at LC_10_ and LC_30_, as well as their mixtures (A_10_D_10_, A_10_D_30_, and A_30_D_30_) after 24 h of treatment (Figure 2A,B). For CAT expression (Figure 2A), the control group exhibited higher mRNA expression levels compared to most treatment groups. Notably, the A_10_ treatment group showed a higher expression level compared to the control group. Although in the other treatments the expression was lower than the control group, the expression level was significantly different in the D_30_, A_10_D_30_, and A_30_D_30_ groups (*F* = 5.23, *p* = 0.0026). As indicated, the mixture-treated group (A_30_D_30_) displayed the lowest expression levels, suggesting a possible synergistic or additive suppressive effect of amitraz and dinotefuran on CAT expression. For SOD expression (Figure 2B), a similar trend was observed. The control group showed the highest mRNA expression levels, while individual amitraz (A_10_, A_30_) and dinotefuran (D_10_, D_30_) treatments led to a reduction in SOD expression. A significant downregulation (*F* = 4.71, *p* = 0.0039) was observed in the combination groups where dinotefuran was used at the higher concentration (A_10_D_30_ and A_30_D_30_), compared to the control, suggesting that a combined exposure may increase oxidative stress effects.

### 2.4. Effects of Amitraz, Diotefuran, and Their Mixture on Honey Bee Hypopharyngeal Glands Morphology

**Figure 3:**
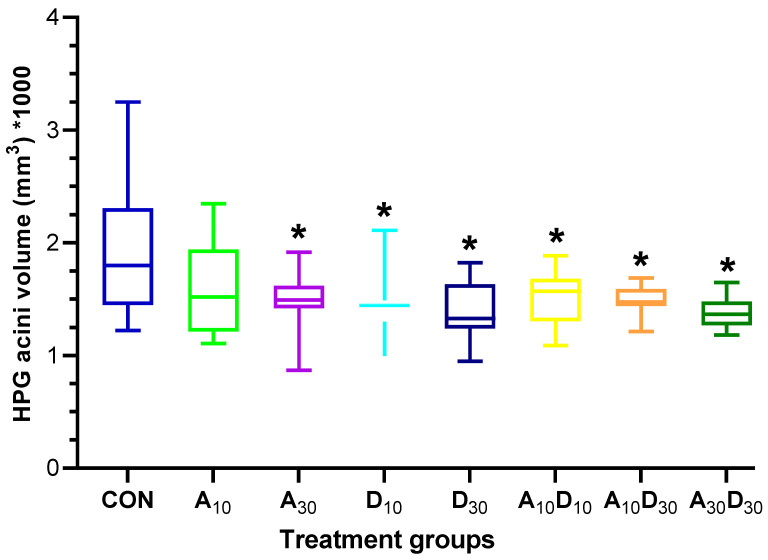
Low lethal effects of amitraz, dinotefuran, and their mixture on hypopharyngeal gland (HPG) morphology in *A. mellifera* workers. Boxplots show the volume of HPG acini (mm^3^ × 1000) in bees exposed to LC_10_ and LC_30_ concentrations of amitraz (A_10_, A_30_), dinotefuran (D_10_, D_30_), and their combinations (A_10_D_10_, A_10_D_30_, A_30_D_30_). The center line represents the median; box edges indicate the first and third quartiles (Q1 and Q3); and whiskers denote the minimum and maximum values. Asterisks indicate significant differences compared to the control group (* *p* < 0.05, Tukey’s HSD test).

Exposure to low lethal concentrations of amitraz and dinotefuran reduced the hypopharyngeal gland (HPG) acini volume in *A. mellifera* workers after seven days of treatment (Figure 3). The control group exhibited the largest acini volume, whereas pesticide exposure led to a general decline. While most treatments caused a significant reduction (*F =* 3.64, *p =* 0.0017), A_10_ was the only group that did not show a statistically significant decrease (*p* = 0.5944). Notably, dinotefuran at D_10_ resulted in a significant decrease, indicating that even lower concentrations of this neonicotinoid negatively impact gland development. Furthermore, the combined exposure groups (A_10_D_10_, A_10_D_30_, A_30_D_30_) exhibited a further reduction in HPG volume, with all combination groups showing significant differences compared to the control (A_10_D_10_ vs. CON, *p* = 0.0334; A_10_D_30_ vs. CON, *p* = 0.0142; A_30_D_30_ vs. CON, *p* = 0.0033). These results suggest that amitraz and dinotefuran may exert additive effects when co-administered, resulting in more pronounced glandular atrophy. The observed impairments in nurse bee physiology highlight potential consequences for brood care and overall colony health.

### 2.5. Effects of Amitraz, Dinotefuran, and Their Mixture on Immune-Related Gene Expression

**Figure 4:**
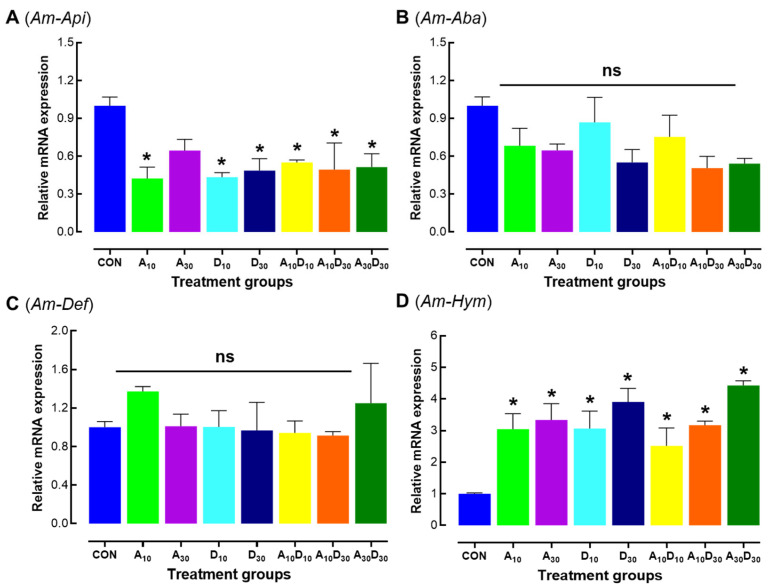
Low lethal effects of amitraz, dinotefuran, and their mixture on immune-related gene expression in newly emerged *Apis mellifera* workers. Relative mRNA expression levels of apidaecin (*Am-Api*) (**A**), abaecin (*Am-Aba*) (**B**), defensin (*Am-Def*) (**C**), and hymenoptaecin (*Am-Hym*) (**D**) were analyzed in honey bees 24 h after exposure to LC_10_ and LC_30_ concentrations of amitraz (A_10_, A_30_), dinotefuran (D_10_, D_30_), and their mixtures (A_10_D_10_, A_10_D_30_, A_30_D_30_). Three independent biological replicates were used per condition. Error bars represent standard deviation of the mean (SD). Asterisks indicate significant differences compared to the control group (* *p* < 0.05, Tukey’s HSD test). “ns” denotes non-significant differences.

*Am-Api* expression was significantly downregulated in all treatment groups compared to the control (*F* = 6.74, *p* = 0.0006), suggesting a suppression of this antimicrobial peptide by amitraz, dinotefuran, and their combinations (Figure 4A). In contrast, *Am-Hym* expression was significantly upregulated in all treatment groups (*F* = 12.55, *p* < 0.0001), with the highest levels observed in the D_30_ and A_30_D_30_ groups, indicating a potential immune response activation (Figure 4D). *Am-Aba* and *Am-Def* expression levels were not significantly affected by insecticide exposure (*Am-Aba*: *F* = 1.19, *p* = 0.3542; *Am-Def*: *F* = 1.15, *p* = 0.3767), suggesting these genes were not substantially altered (Figure 4B,C). These results indicate that while some immune-related genes (*Am-Aba* and *Am-Def*) remained unchanged, others (*Am-Api* and *Am-Hym*) exhibited significant disruptions in expression, reflecting differential immune modulation depending on the gene and treatment type.

### 2.6. Effects of Amitraz, Diotefuran, and Their Mixture on Detoxification-Related Genes Expression

**Figure 5:**
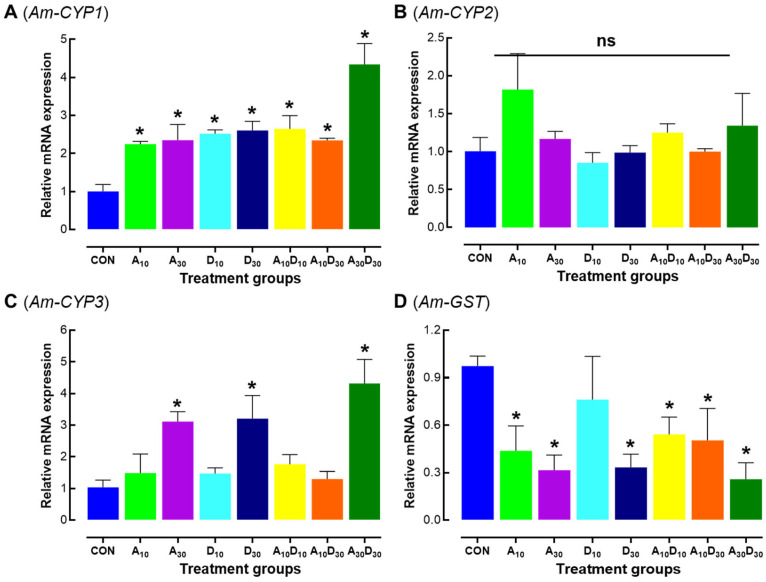
Low lethal effects of amitraz, dinotefuran, and their mixture on detoxification-related gene expression in newly emerged Apis mellifera workers. Relative mRNA expression levels of cytochrome P450 genes (Am-CYP1) (**A**), (Am-CYP2) (**B**), (Am-CYP3) (**C**), and glutathione S-transferase (Am-GST) (**D**) were analyzed in honey bees 24 h after exposure to LC_10_ and LC_30_ concentrations of amitraz (A_10_, A_30_), dinotefuran (D_10_, D_30_), and their mixtures (A_10_D_10_, A_10_D_30_, A_30_D_30_). Three independent biological replicates were used per condition. Error bars represent standard deviation of the mean (SD). Asterisks indicate significant differences compared to the control group (* *p* < 0.05, Tukey’s HSD test). “ns” denotes non-significant differences.

The results showed that *Am-CYP1* expression was significantly upregulated in all treatment groups, with the highest expression observed in the A_30_D_30_ group (*F* = 12.82, *p* < 0.0001; Tukey’s HSD: A_30_D_30_ vs. CON, *p* = 0.0002) (Figure 5A). *Am-CYP2* expression remained unchanged across all treatments (*F* = 1.21, *p* = 0.3422) (Figure 5B), while *Am-CYP3* was significantly upregulated in the high concentrations of both insecticides as well as their mixture (*F* = 8.77, *p* = 0.0002) (Figure 5C), suggesting a stronger induction of specific cytochrome P450 genes at higher insecticide concentrations. In contrast, *Am-GST* expression was significantly downregulated in all treatment groups except D_10_ compared to the control (*F* = 10.31, *p* < 0.0001) (Figure 5D), indicating a potential impairment in glutathione S-transferase activity. These findings suggest that a low lethal exposure to amitraz and dinotefuran differentially affects detoxification-related genes, with cytochrome P450 genes (*Am-CYP1* and *Am-CYP3*) being induced, while *Am-GST* expression is suppressed, potentially altering the detoxification and stress response mechanisms in honey bees.

## 3. Discussion

Honey bees are continuously exposed to environmental stressors, including pesticides used in agriculture and beekeeping, which can compromise their health and survival [6,7]. Among these, neonicotinoid insecticides and acaricides used against *Varroa destructor* are of particular concern due to their potential combined effects [7,14,15,22]. Our findings indicate that dinotefuran poses a significantly higher toxicity risk to honey bees than amitraz (Table 1), emphasizing the need for a more comprehensive risk assessment of pesticide exposure in managed colonies. Given the frequent use of these compounds together, understanding their interactions is crucial for developing safer pest management strategies in apiculture.

The low lethal concentrations of amitraz and dinotefuran (LC_10_ and LC_30_) used in this study were selected based on acute oral bioassays and also correspond to residue levels detected under realistic field conditions. For instance, dinotefuran has been found in honey and pollen at concentrations ranging from 1 to 6 ng/g in agricultural landscapes [35], while amitraz and its metabolites (especially DMF) have been reported in beeswax and brood combs at levels exceeding 100 ng/g in some apiaries [36,37]. Although these values may appear lower than our test concentrations in parts per million (ppm), it is important to note that chronic exposure through ingestion and accumulation in hive matrices can lead to internal concentrations comparable to those tested here. Therefore, the physiological impairments observed in our study may realistically occur under field-relevant conditions, particularly with repeated or cumulative pesticide exposure.

Our findings demonstrate that exposure to amitraz and dinotefuran significantly reduces honey bee survival in a dose-dependent manner, with higher concentrations causing faster mortality (Figure 1A). These results are consistent with previous studies showing that low lethal concentrations of pesticides can decrease honey bee survival [29,38]. Notably, co-exposure to both pesticides exacerbated toxicity, as reflected by the more rapid decline in survival in the combination treatments, particularly A_30_D_30_. This suggests a potential synergistic effect, where amitraz and dinotefuran interact to amplify their toxic impact beyond their individual effects. Such synergistic interactions have been reported before, with some combinations causing higher mortality and enhanced low lethal effects in honey bees [39,40,41,42,43].

Beyond survival, our results indicate that pesticide exposure significantly suppresses vitellogenin (*Am-Vg*) expression, with reductions observed within 24 h and becoming more pronounced after seven days (Figure 1B,C). Since vitellogenin is a key biomarker of honey bee health [44], its downregulation may play a role in reduced survival. This finding aligns with previous reports of vitellogenin downregulation following pesticide exposure [45,46,47,48]. The most pronounced suppression was observed in the A_30_D_30_ group, reinforcing the idea of a synergistic toxic effect. This suppression may be mediated by increased oxidative stress, as pesticide exposure is known to induce reactive oxygen species (ROS) and reduce antioxidant enzyme activity [7,49]. As vitellogenin is essential for regulating oxidative stress, its depletion may heighten bees’ vulnerability to environmental challenges and contribute to increased mortality.

The observed suppression of hypopharyngeal gland morphology and vitellogenin expression may result from pesticide-induced oxidative stress and hormonal disruption. Reactive oxygen species (ROS) accumulation is known to interfere with cellular signaling and protein synthesis in nurse bees, potentially impairing glandular tissue development and metabolic gene regulation. Vitellogenin expression is tightly regulated by endocrine pathways, particularly juvenile hormone (JH) and insulin/IGF-like signaling (IIS), both of which can be disrupted by neonicotinoids and acaricides [44,50]. Thus, it is likely that the combined exposure to amitraz and dinotefuran perturbs these pathways, leading to the physiological impairments observed. Future studies using transcriptomic or proteomic profiling would help confirm these mechanistic links.

Supporting this mechanism, our findings show that exposure to dinotefuran at LC_30_ (D_30_) and its combinations (A_10_D_30_, A_30_D_30_) significantly downregulated catalase (*Am-CAT*) and superoxide dismutase (*Am-SOD*) expression, indicating impaired antioxidant defense (Figure 2). The control group exhibited the highest expression levels, suggesting a stable oxidative balance, whereas the A_30_D_30_ group showed the most pronounced suppression, consistent with previous findings [50,51]. These findings suggest that the synergistic toxicity observed in honey bees exposed to amitraz and dinotefuran is likely driven, at least in part, by oxidative stress-induced damage. The suppression of antioxidant defense mechanisms, along with reduced vitellogenin expression, may disrupt cellular homeostasis and impair immune responses, ultimately increasing mortality. Similar oxidative stress-mediated toxicity has been reported in honey bees exposed to various pesticides, reinforcing oxidative imbalance as a key factor in pesticide-induced honey bee health decline [51,52,53,54]. This aligns with our survival analysis, where the combination treatments led to the most rapid decline in survival, highlighting the potential for amitraz and dinotefuran to act synergistically in exacerbating oxidative stress and physiological decline in honey bees.

The hypopharyngeal glands (HPGs) are vital for honey bee colony maintenance, as they produce royal jelly, essential for larval and queen development [55,56]. The impairment of HPG development can disrupt brood care and contribute to colony decline [57,58,59,60]. Our findings indicate that low lethal exposure to amitraz and dinotefuran reduces acini volume in all treatment groups, with the most significant decline in the A_30_D_30_ group (Figure 3), suggesting a synergistic effect. Similar HPG morphological alterations resulting from insecticide exposure have been reported [58,59,61]. The reduction in gland size may impair nurse bee physiology, resulting in inadequate larval nutrition, weakened brood development, and compromised colony health. These findings underscore the need for further investigations into the long-term consequences of pesticide exposure on honey bee physiology and colony sustainability.

The differential expression of antimicrobial peptides (AMPs) in response to pesticide exposure highlights the complex immune modulation in honey bees. Such insecticides are known to disrupt immune function, thereby increasing colony mortality [62,63,64]. The significant downregulation of apidaecin (*Am-Api*) across all treatments suggests immune suppression (Figure 4A), which may render bees more vulnerable to pathogens. This finding is consistent with previous studies [65,66,67]. Conversely, the upregulation of hymenoptaecin (*Am-Hym*) indicates a potential compensatory immune response to pesticide-induced stress (Figure 4D), whereas the unchanged expression of abaecin (*Am-Aba*) and defensin (*Am-Def*) points to selective immune modulation (Figure 4B,C). These results align with prior studies showing that pesticides can either suppress or stimulate immune responses depending on their mode of action and concentration [38,65,66,68]. Given the critical role of AMPs in pathogen defense, such alterations may have long-term consequences for colony health in pesticide-exposed environments.

Cytochrome P450 (CYP) and glutathione S-transferase (GST) enzymes play vital roles in the detoxification of harmful compounds in honey bees [69]. Our study reveals a significant upregulation of *Am-CYP1* and *Am-CYP3* in bees exposed to insecticides (Figure 5A,C), indicating a strong induction of P450 genes, suggesting that both amitraz and dinotefuran activate detoxification pathways. This is consistent with previous studies highlighting the involvement of P450 enzymes in pesticide metabolism [33,41]. In contrast, *Am-GST* expression was downregulated (Figure 5D), suggesting an impaired GST-mediated detoxification process. This observation is consistent with previous studies on pesticide exposure [31,34,70]. Reduced GST activity may contribute to elevated oxidative stress, as GSTs are involved in detoxifying reactive oxygen species (ROS) [7,70]. The synergistic effect observed in the A_30_D_30_ combination, where both P450 and GST responses were amplified, supports previous research showing that pesticide mixtures can result in heightened toxicity and altered gene expression [31,39]. These findings suggest that exposure to amitraz and dinotefuran disrupts detoxification mechanisms in honey bees, potentially compromising their ability to manage chemical stressors. Alterations in detoxification-related gene expression could impair the bees’ defense systems, underscoring the need for further studies on the long-term effects of pesticide exposure on honey bee health and colony viability [44,53].

The observed synergistic effects of amitraz and dinotefuran may result from interactions occurring at multiple physiological levels. Amitraz acts as an octopamine receptor agonist and may interfere with neural regulation and stress response systems, whereas dinotefuran targets nicotinic acetylcholine receptors. Although the specific interaction between these two compounds remains unclear, previous studies have reported additive neurotoxic effects resulting from co-exposure to cholinergic pesticides [71]. These findings support the hypothesis that combined pesticide exposure may enhance toxicity through the disruption of neural signaling.

## 4. Materials and Methods

### 4.1. Experimental Design

**Figure 6:**
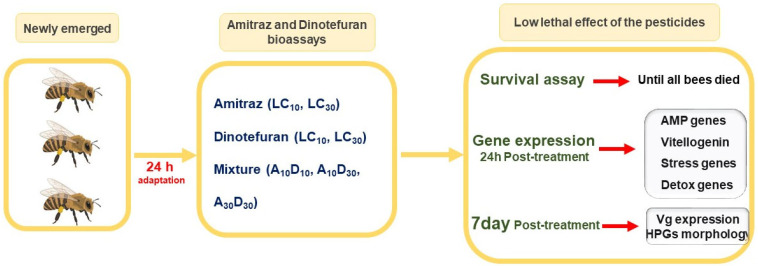
Schematic diagram of the experimental design for evaluating low lethal effects of amitraz and dinotefuran on honey bees.

The experiments were designed to assess the low lethal (LC_10_ and LC_30_) effects of amitraz, dinotefuran, and their combinations on honey bee survival, gene expression, and hypopharyngeal gland morphology (Figure 6). Eight experimental groups were established: control, amitraz at LC_10_ and LC_30_, dinotefuran at LC_10_ and LC_30_, and three combinations (A_10_D_10_, A_10_D_30_, A_30_D_30_). Each group included 10 replicates, with 10 bees per replicate. All experiments were conducted under controlled laboratory conditions (33 ± 0.1 °C, 65 ± 5% RH), with access to 50% sucrose solution and pollen patty. Exposure durations varied by experiment: 48 h for acute toxicity bioassay, until death for survival analysis, 7 days for hypopharyngeal gland dissection, and 24 h for gene expression analysis. Details of each experimental procedure are described in the following subsections.

### 4.2. Honey Bees

Newly emerged worker bees were collected from colonies located at the experimental apiary of Gyeongkuk National University, Andong, Republic of Korea. Bees were collected from three distinct colonies. Individuals from each colony were randomly and equally distributed across all treatment groups to ensure a balanced biological replication. The experiments, including bioassay and survival assays, were initiated in September. Bees were placed in an insect breeding cap (120 × 80 mm, SPL-TDS-ISBDJ, Pocheon, Republic of Korea) following a 24 h adaptation period. The bees were reared under controlled conditions at a temperature of 33 ± 0.1 °C and relative humidity of 65 ± 5. Under the normal condition, bees were provided with a 50% sucrose solution (*w*/*v*), which was refreshed daily. A pollen patty was also provided to ensure adequate protein intake during the experimental period.

### 4.3. Chemicals

Amitraz (20% emulsion, formulation, Arysta Life Science, Cary, NC, USA), dinotefuran (10% liquid formulation, Yong-Il Chemical Co., Ltd., Seoul, Republic of Korea), and acetone (purity 99.5%; CAS No. 67-64-1; Daejung, Republic of Korea) were purchased and used in this study.

### 4.4. Acute Toxicity Bioassay

An acute oral toxicity assay was conducted to evaluate the effects of amitraz and dinotefuran on honey bee workers, following modified procedures from Medrzycki et al. (2013) [72]. For this purpose, newly emerged bees were gently anesthetized with carbon dioxide to facilitate handling, as previously outlined by Human et al. (2013) [73]. Groups of ten bees were transferred into individual rearing containers and subjected to a 2 h starvation period under controlled laboratory conditions (33 ± 1 °C; 65 ± 5% RH) prior to treatment. Pesticide stock solutions were prepared by dissolving the formulations in 1 mL of analytical-grade acetone. These solutions were subsequently diluted with 50% sucrose to obtain the desired test concentrations. Six concentrations were serially diluted in a 50% (*w*/*v*) sucrose solution. The test concentrations for amitraz were 1000, 500, 250, 125, 62.5, and 31.2 ppm, while those for dinotefuran were 50, 25, 12.5, 6.25, 3.125, and 1.562 ppm. After pesticide administration, bees were provided only with a 50% (*w*/*v*) sucrose solution and distilled water, which were refreshed daily throughout the experimental period. Control honey bees only received a 50% (*w*/*v*) sucrose solution and distilled water. Each treatment and control group consisted of five replicates, each containing ten bees. Honey bee mortality was recorded 48 h after pesticide administration. Honey bees were considered as dead when a complete motionlessness was observed upon a gentle probe with a fine brush.

### 4.5. Survival and Longevity Experiment

Briefly, bees were fed a 50% (*w*/*v*) sucrose solution containing the insecticides—amitraz and dinotefuran—at LC_10_ and LC_30_ concentrations, as well as their mixtures (prepared by combining equal volumes of each concentration; 50:50 *v*/*v*) for 4 h. They were then switched to a 50% (*w*/*v*) sucrose solution. The control groups were fed a 50% sucrose solution throughout their lifetimes. The solutions were refreshed daily, and mortality was recorded daily until all bees had died. A total of eight treatment groups were included: control, amitraz LC_10_ and LC_30_, dinotefuran LC_10_ and LC_30_, and three combinations (A_10_D_10_, A_10_D_30_, A_30_D_30_). Each group included 10 replicates, with 10 bees per replicate.

### 4.6. Measurement of Secretion Glands

For the analysis of hypopharyngeal gland (HPG) morphology, the heads of treated honey bee workers were carefully dissected under a stereomicroscope to isolate paired HPGs, following established protocols [74] (Figure S1). Bees used for this assay were raised separately under the same experimental treatments described for the survival and gene expression studies. After seven days of exposure, ten bees per treatment group were selected for microscopic assessment. From each dissected HPG, five acini were randomly chosen for morphometric measurement. The maximum length and width of each acinus were recorded using a compound microscope equipped with a digital imaging system (BX53F, Olympus, Tokyo, Japan; IMTcamCCD5 plus, IMT i-Solution, Vancouver, BC, Canada). Gland volume was then estimated using the following formula:

$$\mathrm{Volume}=\frac{4}{3}\pi \times {\left.\left(\frac{a+b}{4}\right.\right)}^{3}$$

where “*a*” is the maximum length and *b* is the maximum width of the acini of glands, and π = 3.14 [74].

### 4.7. RNA Extraction and RT-qPCR

In this study, the expression of the following genes was assessed: vitellogenin (*Am-Vg*); antioxidant-related genes including catalase (*Am-CAT*) and superoxide dismutase (Am-SOD); antimicrobial peptide genes including apidaecin (*Am-Api*), abaecin (*Am-Aba*), defensin (*Am-Def*), and hymenoptaecin (*Am-Hym*); and detoxification-related genes including cytochrome P450s (*Am-CYP1*, *Am-CYP2*, *Am-CYP3*) and glutathione S-transferase (*Am-GST*). Actin (*Am-actin*) was used as the reference gene. For this experiment, newly emerged bees were reared and treated separately under the same low lethal pesticide conditions described previously. To assess gene expression following low lethal pesticide exposure, worker bees were sampled post-treatment and rapidly frozen in liquid nitrogen. Total RNA was isolated from whole bodies of adult bees using TRIzol^®^ reagent (Invitrogen, Carlsbad, CA, USA), following the manufacturer’s guidelines. RNA quality and concentration were assessed using a NanoDrop spectrophotometer (Thermo Fisher Scientific, Waltham, MA, USA). Subsequently, 100 ng of total RNA was reverse transcribed into cDNA using the RT-premix kit (Intron Biotechnology, Seoul, Republic of Korea). Quantitative real-time PCR (qPCR) was conducted with SYBR Green master mix (Toyobo, Osaka, Japan) on a StepOnePlus Real-Time PCR System (Applied Biosystems, Singapore). Each 20 µL reaction included 10 pmol of specific primers (listed in Table S1) and 100 ng of cDNA. Thermal cycling conditions involved an initial activation step at 94 °C for 5 min, followed by 40 cycles of 94 °C for 20 s, primer-specific annealing (see Table S1) for 20 s, and extension at 72 °C for 20 s. Relative gene expression was normalized against actin (Am-actin) as the internal control, and data were analyzed using the 2^−ΔΔCT^ method [75]. All treatments were tested in three biological replicates prepared independently.

### 4.8. Data Analysis

The acute oral exposure against adult honey bees of each insecticide’s regression lines (48-h LC50), 95% confidence limits, and χ^2^ were calculated for toxicity test responses by a Probit analysis using SPSS software (Version 16.0 for Windows, SPSS Inc., Chicago, IL, USA). An analysis of variance (ANOVA) followed by the post hoc Tukey’s test were conducted for statistical analysis using GraphPad Prism version 8.2.0 (La Jolla, CA, USA). A Kaplan–Meier survival analysis was performed to evaluate worker bee survival across treatments, and survival curves were compared using the log-rank (Mantel–Cox) test. This analysis was conducted using GraphPad Prism version 8.2.0, and statistical significance was set at *p* < 0.05.

## 5. Conclusions

In conclusion, this study demonstrates that both amitraz and dinotefuran significantly affect honey bee survival, physiological health, and immune function. While dinotefuran exhibits higher toxicity than amitraz, their combination results in even more severe adverse effects. These synergistic impacts, particularly at higher concentrations, include reduced survival, disrupted expression of vitellogenin (*Am-Vg*) and antioxidant enzymes (*Am-CAT*, *Am-SOD*), damage to hypopharyngeal glands, and alterations in immune (*Am-Api*, *Am-Hym*) and detoxification-related genes. Notably, *Am-CYP1* was markedly upregulated, while *Am-GST* was significantly downregulated. Given the widespread use of these pesticides in both agriculture and *Varroa* mite control, this study underscores the urgent need for risk assessments and regulatory measures to mitigate their impact on honey bee health and agricultural ecosystems. Given the widespread use of these pesticides in both agriculture and *Varroa* mite control, this study underscores the urgent need for risk assessments and regulatory measures to mitigate their impact on honey bee health and agricultural ecosystems. Future research should investigate long-term and colony-level impacts of these pesticide combinations under field conditions, as well as potential recovery mechanisms and protective interventions to support bee health.

## Figures and Tables

**Table 1:** Oral low (LC_10_, LC_30_) and median (LC_50_) lethal concentrations (ppm) of amitraz and dinotefuran to worker honey bee, *Apis mellifera*.

Treatment	LC_10_^a^ (95% CL ^b^)	LC_30_ (95% CL)	LC_50_ (95% CL)	Slope ± SE	*X* ^2^
Amitraz	179.0 (88.6–253.1)	301.4 (199.4–399.2)	432.3 (320.6–596.9)	3.3 ± 0.4	46.4
Dinotefuran	1.027 (0.082–2.118)	2.088 (0.426–3.464)	3.413 (1.274–5.093)	2.5 ± 0.4	45.1

^a^ LC_10_ and LC_30_ shown in Table 1 were used as low lethal concentrations for subsequent analyses. ^b^ Confidence limits. Note all LC’s are 48 h after treatments.

## Data Availability

All data generated or analyzed during this study are included in this published article and its Supplementary Information File. The genome sequence datasets generated and/or analyzed during the current study are available in GenBank repository using accession numbers in Table S2.

## Supplementary Materials

The supporting information can be downloaded at: https://www.mdpi.com/article/10.3390/ijms26146850/s1.
