# Supplementary material for: Synergistic Effects of Amitraz and Dinotefuran on Honey Bee Health: Impacts on Survival, Gene Expression, and Hypopharyngeal Gland Morphology
Source: Int J Mol Sci. 2025 Jul 17;26(14):6850. doi: 10.3390/ijms26146850 (PMC12296131; doi:10.3390/ijms26146850)
Supplement: Supplementary file 1 [file ijms-26-06850-s001.zip › ijms-3699574-supplementary.pdf]

Supplementary data

Table S1. Primers used in this study

Genes	Orientation	Sequence (5′ - 3′)	Uses	Annealing temperature (°C)
β-Actin	Forward	CTAGCACCATCCACCATGAAA	RT-qPCR	52.0
	Reverse	AGGTGGACAAAGAAGCAAGAA		
CYP1	Forward	GCGTCATTGGAGCGTTACTGTTCG	RT-qPCR	52.0
	Reverse	AATTGTTTCGTTTTCTCGGCCAGTG		
CYP2	Forward	GCAAGAGCGTGGACCCGATG	RT-qPCR	52.0
	Reverse	AGGTGAAGGTGGGCGAAAGCA		
CYP3	Forward	TGAAACTCATGACCGAGACG	RT-qPCR	52.0
	Reverse	AAAATTTGGGCCGCTAATAAA		
GST	Forward	TGCATATGCTGGCATTGATT	RT-qPCR	52.0
	Reverse	TCCTCGCCAAGTATCTTGCT		
Vitellogenin	Forward	GCAGAATACATGGACGGTGT	RT-qPCR	52.0
	Reverse	GAACAGTCTTCGGAAGCTTG		
Catalase	Forward	GTCTTGGCCCAAACAATCTG	RT-qPCR	52.0
	Reverse	CATTCTCTAGGCCCACCAAA		
Superoxide dismutase	Forward	AAGCAGTGTGCGTTCTTCAGGGT	RT-qPCR	52.0
	Reverse	TCACGGAATTGGTACTCTCCGGTT		
Abaecin	Forward	CAGCATTCGCATACGTACCA	RT-qPCR	52.0
	Reverse	GACCAGGAAACGTTGGAAAC		
Defensin	Forward	TGCGCTGCTAACTGTCTCAG	RT-qPCR	52.0
	Reverse	AATGGCACTTAACCGAAACG		
Hymenoptaecin	Forward	CTCTTCTGTGCCGTTGCATA	RT-qPCR	52.0
	Reverse	GCGTCTCCTGTCATTCCATT		
Apidaecin	Forward	TTTTGCCTTAGCAATTCTTGTTG	RT-qPCR	52.0
	Reverse	GTAGGTCGAGTAGGCGGATCT		

Table S2. GenBank accession numbers of genes used in this study

Gene name	Acronym	Accession number
Vitellogenin	*Am-Vg*	NM_001011578
Hymenoptaecin	*Am-Hym*	U15956
Defensin	*Am-Def*	U15955
Abaecin	*Am-Aba*	NM_001011617
Apidaecin	*Am-Api*	X72575.1
Catalase	*Am-CAT*	NM_001178069
Superoxide dismutase	*Am-SOD*	NM_001178027
CYPS63	*Am-CYP1*	GB16447
CYP9Q2	*Am-CYP2*	ON649465.1
CYP6AS	*Am-CYP3*	GB17434
Glutathione S-transferase	*Am-GST*	GB18045
Actin	*Am-Actin*	NM_001185146

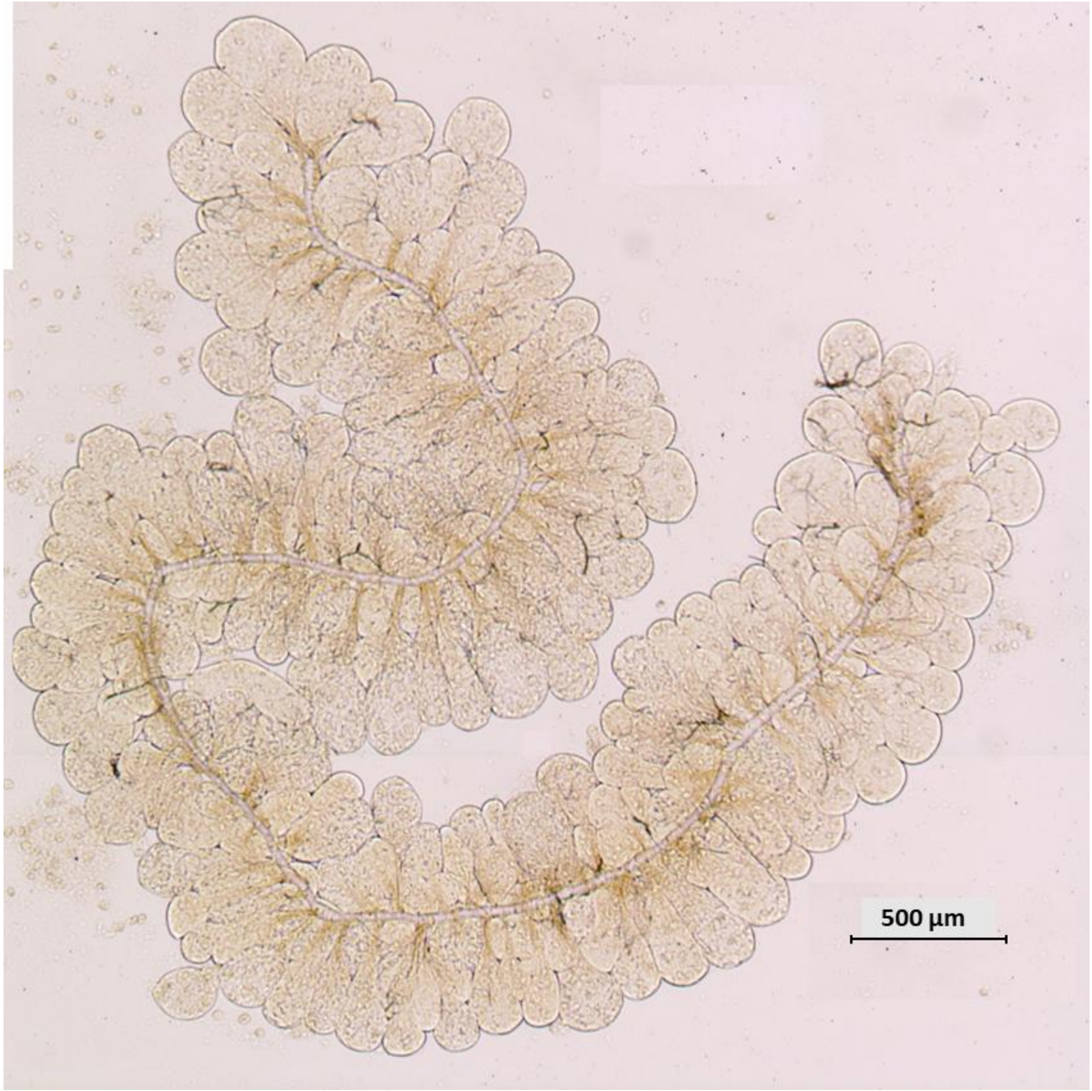

Fig S1. Hypopharyngeal gland (HPG) acini on day 7 of caged honey bees.
